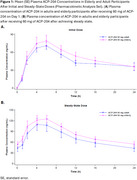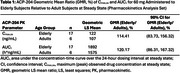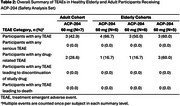# Pharmacokinetics in Healthy Adult and Elderly Patients of ACP‐204, a Novel 5‐HT_2A_ Receptor Selective Antagonist/Inverse Agonist

**DOI:** 10.1002/alz70859_105732

**Published:** 2025-12-26

**Authors:** Mona Darwish, Xiaoshu Feng, Bryan Dirks, Brian Raether, Sanjeev S Pathak

**Affiliations:** ^1^ Acadia Pharmaceuticals Inc., Princeton, NJ USA

## Abstract

**Background:**

ACP‐204, a potent inverse agonist/antagonist of 5‐HT_2A_ receptors, is under investigation for the treatment of Alzheimer’s disease psychosis (ADP). Frail elderly patients with ADP are vulnerable to adverse effects of antipsychotics and potential age‐related differences in drug pharmacokinetics (PK) and pharmacodynamics. We investigated the PK, safety, and tolerability of ACP‐204 in healthy elderly and adult participants at the proposed clinical dose (60 mg).

**Method:**

In this single‐center, phase 1, double‐blind, multiple ascending dose study, healthy elderly and adult participants were randomized to receive ACP‐204 oral doses up to 60 mg and 120 mg, respectively. Steady‐state plasma samples were collected daily up to 10 days of dosing. The maximum observed drug concentration at steady state (C_max‐ss_) and the area under the concentration‐time curve over the 24‐h dosing interval at steady state (AUC_τ_) were compared. Due to demonstrated linearity data, the 60‐mg dose was selected as a representative of the PK parameters for the comparison. Safety was evaluated at each visit and during follow‐ups.

**Result:**

Seven healthy adult participants in 1 cohort and 17 elderly participants in 3 cohorts received 60 mg of ACP‐204. Data from the elderly were pooled for steady‐state PK evaluation. Median T_max‐ss_ was ∼5‐6 h in elderly and ∼4‐6 h in adult cohorts. Decline from peak was monophasic, with mean t_1/2_ of 19.8 and 17.8 h, respectively. In elderly participants, C_max‐ss_ and AUC_τ_ were higher than those of the adults by 1.14‐fold and 1.20‐fold, respectively. The geometric least squares mean of the AUC_τ_ for elderly vs adult participants was 1892 h*ng/mL vs 1575 h*ng/mL at 60 mg. The geometric mean ratio (elderly/adults) of the AUC_τ_ was 120.17% (90% CI, 86.31%, 167.32%) (Table 1). Plasma ACP‐204 concentrations are shown in Figure 1. Safety profiles were similar between age groups (Table 2).

**Conclusion:**

The PK profiles were qualitatively similar over the studied dose ranges in healthy elderly and adult participants with minimal difference in exposure parameters 14% and 20% for C_max,ss_ and AUC _τ_, respectively. ACP‐204 was generally safe and well tolerated across age groups, with no unexpected safety findings.